# Effects of natural antimicrobials with modified atmosphere packaging on the growth kinetics of *Listeria monocytogenes* in ravioli at various temperatures

**DOI:** 10.1111/jfs.12392

**Published:** 2017-08-25

**Authors:** Eun Young Ro, Geun Su Kim, Do Young Kwon, Young Min Park, Sang Woo Cho, Sang Yun Lee, Ik Hyun Yeo, Ki Sun Yoon

**Affiliations:** ^1^ R&D Center for Food Technology Pulmuone Co., Ltd Seoul South Korea; ^2^ Department of Food and Nutrition Kyung Hee University Seoul South Korea

## Abstract

The objective of this study was to investigate the antimicrobial effects of cultured sugar/vinegar (CSV) blend and nisin to control the risk of *Listeria monocytogenes* in ready to cook (RTC) ravioli. Ravioli dough was prepared with 0.1, 0.3, 0.5, 1% CSV blend and 0.1, 0.2, and 0.3% nisin. Inoculated spinach or artichoke raviolis with 2.0 ± 0.5 log cfu/g of *L. monocytogenes* were packed aerobically or using modified atmosphere packaging (MAP), and then stored at 4, 10, 17, and 24 °C for 60 days. Growth kinetic parameters of the observed data fit well to the Baranyi equation. Ravioli with spinach filling materials yielded a higher risk than that with artichoke. *L. monocytogenes* was able to survive in ravioli with artichoke, but did not grow. The addition of 1% CSV blend or 0.3% nisin in spinach ravioli with the combination of MAP effectively controlled the growth of *L. monocytogenes* at the temperature below 10 °C. The organoleptic quality of spinach ravioli was not also affected by the application of 1% CSV blend. Therefore, the CSV blend can be recommended to improve the microbial safety and quality of natural RTC ravioli at retail market.

**Practical applications:**

The risk of ravioli was affected by the filling materials of ravioli at retail market. Addition of 1% cultured sugar/vinegar blend in dough substantially contributes to the extension of shelf‐life of MAP spinach raviolis. classification and regression tree analysis results indicate that refrigeration temperature is the main control factor to affect lag time and growth rate, while packaging method is critical for maximum population density.

## 1 INTRODUCTION

With the increase of consumer demand for foods with more convenient, affordable and easy‐to‐cook portions, a wide range of new products, such as chilled/filled pasta, including raviolis, have been introduced to the market. According to the Euromonitor International (2012), the market for chilled/filled pasta, such as raviolis, grows faster than other product markets with the total sales worth U.S. $348.8 million by 2016. As consumption of these foods increases, safety of refrigerated/filled raviolis at retail market should be ensured. Manufacturing environment has been linked to many cases of foodborne listeriosis and frozen ravioli with spinach was recalled for potential *Listeria* contamination (Food Safety News, 2015). Although the chilled products are cool‐processed, *L. monocytogenes* is frequently able to survive and grow even under refrigeration temperature (Larson, Johnson, & Nelson, 1999). *L. monocytogenes* is widely distributed in nature and causes serious concerns to food industries and regulatory agencies due to its high hospitalization rate (94%) and fatality rate (Warriner & Namvar, 2009).

Several methods are being employed to control *Listeria* in food industries, mostly by adapting antimicrobials. In recent years, consumers are seeking natural or organic foods (Organic Trade Association, 2015). Around 73% of all U.S. households occasionally purchase organic foods (Organic Trade Association, 2009). Thus, to emphasize the safety of used ingredients, producers are seeking clean‐label alternative ingredients with less “chemical sounding” names. Cultured sugar and vinegar blend (CSV blend, Purac verdad NV55), a natural alternative to lactic acid salt compound, is generally regarded as safe and can be added to food products during processing. A natural blend of CSV consists of fermentation with specially curated cultures, such as sugars, organic acids, peptides, and aromas. The reagent is designed to have a high level of *Listeria* control (Purac, 2013). Glass and Sindelar (2010) and Sullivan et al. (2012) reported that the CSV blend inhibited the growth of *L. monocytogenes* in meat products. Recently, antimicrobial effect of 3% CSV blend on behavior of *Campylobacter jejuni* and *Salmonella* Typhimurium in chicken breast was also reported (Park, Hong, & Yoon, 2014).

In the present study, we investigated the antimicrobial effect of CSV blend on the growth control of *L. monocytogenes* in raviolis filled with spinach or artichoke and compared to that of nisin. Combined effects of modified atmosphere packaging (MAP) and storage temperature as hurdle techniques at retail market were also evaluated.

## 2 MATERIALS AND METHODS

### 2.1 Bacterial culture for inoculation study

*L. monocytogenes* strains (ATCC 19111, ATCC 19115, and ATCC 15313) were purchased from the Korean Culture Center of Microorganisms (KCCM, Seoul, Korea) and maintained at −80 °C with tryptic soy broth (TSB, Difco, Sparks, MD) with 0.6% yeast extract (TSBYE) (Oxoid, Basingstoke, United Kingdom) in beads. For each experiment, each stock culture was thawed and inoculated in 10 ml of TSBYE, followed by incubation at 37 °C for 24 hr. Viable cell counts of *L. monocytogenes* ranged between 8.5 and 9.0 log cfu/ml after incubation. An aliquot of 1 ml of the initial culture in stationary phase was transferred into 9 ml of 0.85% NaCl, which was serially diluted. A mixture of three *L. monocytogenes* strains was prepared before inoculation into the ravioli samples.

### 2.2 Preparation and inoculation of ravioli

As antimicrobial agents for ravioli dough, a natural blend of CSV (Purac verdad NV55) and nisin were purchased from Purac (Lincolnshire, IL) and Sigma‐Aldrich (St. Louis, MO), respectively. Imported durum semolina flour (Miller milling company, MN) was purchased from samjinFS (Kimpo‐si, Gyeonggi‐do, Korea), and then ravioli dough was prepared with flour, water, and egg (7:2:1 ratio) by an electronic mixer (K45SS, Kitchen aid, MI).

Spinach or artichoke base materials were provided from Giloy ravioli manufacturer (Pulmuone Co., CA). The CSV blend was uniformly added to the spinach or artichoke ravioli dough at the concentration of 0.1, 0.3, 0.5, or 1%, while nisin was added to only spinach ravioli dough at the concentrations of 0.1, 0.2, or 0.3%. Control was prepared without antimicrobial agents. The dough samples were formed into rectangle ravioli shape (8.5 ± 0.5 g) and then filled with 18 g of spinach or artichoke base materials. The prepared raviolis were then heated at 121 °C for 10 min to remove any background microflora. After cooling, the raviolis were transferred into petri dishes (9 × 2 cm). The surface of each ravioli was uniformly inoculated with 0.1 ml of the diluted cultures of *L. monocytogenes* mixture using a sterile repeater pipette to reach the target inoculation levels (2.0 ± 0.5 log cfu/g). The inoculated spinach raviolis were packaged aerobically or in modified atmosphere with gas mixture (0% O_2_, 0.3% CO_2_, and N_2_ 99.7%) (Composite Deoxidation Desiccant, Lipmen, Incheonm, Korea). The inoculated artichoke raviolis were also packed under MAP. Gas‐tight wrapper type bags filled with a deoxidizer (PPEC Eumsung Fresh Noodle Co., Ltd., Eumsung, Korea) were used for MAP. The packaged samples were separately stored at 4, 10, 17, and 24 °C for 60 days.

### 2.3 Enumeration of *L. monocytogenes*

At the specific time intervals during storage, each sample was homogenized (Bag‐Mixer 400, Interscience, Paris, France) in 90 ml of 0.85% NaCl (vol/vol) 2 min. One milliliter of the homogenized sample was diluted with 9 ml of 0.85% NaCl (vol/vol) and 0.1 ml aliquots of two dilutions of each sample was spiral plated (Automatic spiral plater, Interscience, Saint Nom, France) in duplicate on PALCAM agar (Oxoid, Basingstoke, United Kingdom) for *L. monocytogenes* and then incubated aerobically at 36 °C for 48 hr. The colonies on the duplicate plates of each sample were counted by an automated colony counter (Scan 300, Interscience, Saint Nom, France). The results were expressed as log cfu/g and each experiment was repeated twice.

### 2.4 Growth kinetics of primary modeling for *L. monocytogenes* in ravioli

Growth representing viable cell counts (log cfu/g) of *L. monocytogenes* as a function of time was iteratively fit to the Baranyi equation using the DM Fit 3.5 curve‐fitting program (Institute of Food Research, Norwich, United Kingdom). The equation used was as follows (Baranyi & Roberts, 1994):

(1) $$\begin{array}{c} y=y_{0}+\frac{\mu_{\max}}{\ln(10)}A-\frac{1}{\ln(10)}\ln\left[1+\frac{e^{\mu_{\max^{A}}}-1}{10^{\left(y_{\max}-y_{0}\right)}}\right]\\ A=t+\frac{1}{\mu_{\max}}\ln(\frac{e^{\mu_{\max}t}+q_{0}}{1+q_{0}})\\ t_{\text{lag}}=\frac{\ln(1+\frac{1}{q_{0}})}{\mu_{\max}} \end{array}$$

where *y* is the logarithm of the cell numbers (log cfu/g), *y*_0_ is the initial cell number, *y*_max_ is the final cell number, *A* is the time variable, μ_max_ is the specific growth rate (SGR; log per day), *q*_0_ is the physiological state of the inoculum; *t*_lag_ is the lag time (LT); and *t* is the sampling time. The goodness of fit of the data was evaluated based on the coefficient of determination (*R* ^2^). Three parameters, namely, LT, SGR, and maximum population density (MPD) were calculated from the equations described by Baranyi and Roberts (1994) and used for evaluation criteria.

### 2.5 Secondary modeling for the effect of temperature on the growth kinetics of *L. monocytogenes*

Response surface equations as a function of temperature and the concentration of the CSV blend were developed for LT, SGR, and MPD of *L. monocytogenes* in spinach ravioli with MAP by multiple regressions using the SAS (V 9.3) General Linear Models Procedure:

(2) $$\ln y=\;a_{0}+\;a_{1}A+\;a_{2}B+a_{3}A\times B+\;a_{4}A\times A+\;a_{5}B\times B+ɛ$$

where *ln y* is the natural logarithm of the modeled growth parameters (LT, SGR, and MPD), *A* is the temperature, *B* is the concentration of the CSV blend, *a*_0_–*a*_5_ are regression coefficients, and *e* is the random error.

### 2.6 Sensory evaluation

A triangle test was performed to determine whether a significant flavor change occurred in spinach ravioli with 1% CSV blend. The minimum number of panelist needed for the test was determined from the table of significant test for triangle test (Kim, Kim, Sung, & Lee, 1993). To be >95% certain (β = 0.05 and α = 0.05) that no more than 50% (*p*_d_ = 50%) of consumers would be able to detect a difference if CSV the blend was applied to spinach ravioli, the required number of panelists is n = 19. In the present study, 40 trained panelists from the department of research and development at Pulmuone Co., Ltd. participated in sensory evaluation. Spinach ravioli with 1% CSV blend were compared with two controls without antimicrobial treatment. Three samples were randomly numbered with 3‐digit codes and placed in a random order. Two of three samples were identical and the other was different. The panelists were asked to state which product they believed was the odd one, which was different based on appearance, smell, taste, etc. Testing was conducted in individual booths in the Pulmuone R&D center. The panelists were equally spaced throughout the room and instructed not to speak to one another during the test.

### 2.7 Statistical analysis

Three growth kinetics parameters for *L. monocytogenes*‐namely, SGR, LT, and MPD were used to analyze the data. ANOVA model was applied to analyze the effect of multiple treatments for each of the factors: temperature (4, 10, 17, 24 °C) and concentration of antimicrobials (CVS blend: 0.1, 0.3, 0.5, or 1%, nisin: 0.1, 0.2, or 0.3%). For each ANOVA, complete randomized block design was used by setting the other factor combinations as a block using statistical analysis system SAS V 9.3 (SAS Institute Inc., Cary, NC). R package (version 3.1.2) was also used for the classification and regression tree (CART) (Breiman, Friedman, Stone, & Olshen, 1984) analysis. The CART analysis was applied to visualize the suggested hierarchy of variables with respect to the three measured parameters (SGR, LT, and MPD). The CART analysis generates a tree‐like structure by the set of decision points yielding partitions of the original group. The more important variables to affect the response, the higher the position on the tree they have as decision points.

## 3 RESULTS AND DISCUSSION

### 3.1 Comparison of the growth kinetics of *L. monocytogenes* by the kind of ravioli, antimicrobial agent, and packaging

The growth curves of *L. monocytogenes* in both spinach and artichoke ravioli for 60 days well fitted to the Baranyi model. The mean values of the growth kinetics of *L. monocytogenes* were compared according to the kind of raviolis (spinach versus artichoke), antimicrobial agents (CSV blend versus nisin), and packaging (modified atmosphere versus aerobic) (Table 1). There was a higher growth potential of *L. monocytogenes* in spinach ravioli as compared to artichoke ravioli. Although no significant differences were observed in the SGR and LT values, the MPD values were differed significantly between spinach (5.420 log cfu/g) and artichoke raviolis (4.569 log cfu/g). These results indicate that the different filling in ravioli affects the level of stationary phase of *L. monocytogenes* and ravioli with spinach filling has a higher risk than that with artichoke filling. *L. monocytogenes* in artichoke was able to survive, but did not grow, with remaining counts at the initial concentration (around 4.5 log cfu/g) during the storage period (Sanz, Giménez, & Olarte, 2003). In previous research, extracts of artichoke exhibited antimicrobial activity against bacteria species, yeasts, and molds (Emanuel, Adrian, Sultana, & Svetlana, 2011; Ionescu et al., 2013; Zhu, Zhang, & Lo, 2004). 7.7% contamination rate of *L. monocytogenes* in spinach was reported, whereas the pathogen was not isolated from artichokes (Cordano & Jacquet, 2009). Other reports also emphasized the hazard of spinach as a food ingredient for humans. Specifically, Pingulkar, Kamat, and Bongirwar (2001) and Yolanda Moreno et al. (2012) reported that the higher percentage of *L. monocytogenes* positive was detected in spinach samples. *L. monocytogenes* prevalence under such environment was attributed to the cross‐contamination and ability of growth even at refrigeration, as well as to ambient temperature of processed foods (Beuchat, 1996; Wilks, Michels, & Keevil, 2006). The population of *L. monocytogenes* increased from 2.4 to 8.8 log cfu/ml in autoclaved spinach powder cultures at 30 °C, indicating that spinach products provide a good environment for the growth of *L. monocytogenes* (Babic, Watada, & Buta, 1997).

**Table 1:** Comparison of the growth kinetics of *L. monocytogenes* according to the kind of ravioli, antimicrobial agent, and packaging

	Ravioli^a^		Antimicrobial^b^		Packaging^c^	
	Spinach	Artichoke	CSV	Nisin	MAP	Aerobic
SGR	0.568 ± 0.650	0.572 ± 0.836	0.949 ± 00.827	0.854 ± 00.834	0.582 ± 0.627^*^	1.005 ± 0.845
LT	17.636 ± 18.459	8.779 ± 9.456	7.765 ± 11.084	7.458 ± 10.018	15.545 ± 15.766 ^*^	2.510 ± 2.356
MPD	5.420 ± 1.893 ^*^	4.569 ± 1.542	7.434 ± 01.603	6.819 ± 01.866	5.682 ± 1.832^*^	7.507 ± 1.454

LT = lag time (day); SGR = specific growth rate (log/day); MPD = maximum population density (log cfu/g).

^a^Modified packaged ravioli with CSV blend at 0, 0.1, 0.3, 0.5, and 1% was compared.

^b^CSV blend and nisin at 0.1 and 0.3% was compared in spinach ravioli.

^c^Only spinach ravioli treated CSV blend was compared.

*Significant differences between the mean values of growth kinetics were determined by *t* test (*p* < .05).

In addition, antimicrobial effects of 0.1% CSV blend and 0.3% nisin on the control of *L. monocytogenes* growth in spinach raviolis were compared. No significant differences in the LT, SGR, and MPD values were observed between 0.1% CSV blend and 0.3% nisin. Despite this comparable activity, the CSV blend has its advantage of allowing “organic” label. Nisin, which is derived from milk bacteria *Streptococcus lactis* spp., has an effect on controlling a wide range of gram‐positive organisms, including: *Listeria* spp., *Bacillus* spp., and *Clostridium* spp., and their spores (Bruno & Montville, 1993). Although the use of bacteriocins is a novel approach in eliminating or controlling *L. monocytogenes* in food, the development of resistant strains remains the main concern and limits this use as a biopreservative (Kaur et al., 2011). In addition, nisin is not currently approved as an allowable ingredient in organic food and is expensive to use. Therefore, if two reagents are considered as comparable antimicrobial activity, food industry could benefit more by adapting the CSV blend in ravioli products, rather than nisin. The CSV blend is a mixture of natural ferments derived from carefully selected food cultures, including organic acids, sugars, and peptides. Organic acids, such as propionic, citric, lactic acid, exhibited significant antibacterial effects against *L. monocytogenes* (McDonnell, Glass, & Sindelar, 2013; Norhana, Poole, Deeth, & Dykes, 2012; Park et al., 2011). Organic acids are favored due to their intracellular permeability from inherent hydrophobicity and the resulting interactions with the cellular lipid bilayer. This would result in cell damage, death, or exhaustion due to expenditure of energy and resources to exclude the acid.

Response of a microbial cell depends on the nature and amount of direct inhibitors and the influence of the environment (Caillet, Millette, Salmieri, & Lacroix, 2006; Kostaki, Giatrakou, Savvaidis, & Kontominas, 2009). One of the factors contributing to the microbicidal effectiveness of antimicrobials is the nature of the atmosphere in contact with the target cells. It is well known that the composition (e.g., O_2_, N_2_, and CO_2_) of modified atmosphere systems can be an effective means to restrict or inhibit the growth of aerobic spoilage organisms of perishable foods, as well as to maintain the visual quality of products (Narasimha Rao & Sachindra, 2002; Stanbridge & Davies, 1998). In this study, we compared the growth kinetics of *L. monocytogenes* in spinach ravioli under two packaging conditions (MA and aerobic). A significant effect of MAP on growth kinetics (*p* < .05) was observed as compared to aerobic packing. Under the same concentrations of the CSV blend, inhibitory power was improved when extra hurdle of MAP was applied. SGR of *L. monocytogenes* under MAP was approximately a half (0.582 log/day) of that under aerobic condition (1.005 log/day, Table 1). Moreover, the LT in spinach ravioli with MAP was significantly extended up to 15.5 days, as compared to 2.5 days in aerobic packaged spinach ravioli. MPD was also lower in spinach ravioli with MAP (5.68 log cfu/g) than with aerobic packaging (7.51 log cfu/g). The combined treatment of low oxygen and high concentration of CO_2_ or N_2_, can provide adequate suppression of the growth of *L. monocytogenes* (Kostaki et al., 2009). According to Whitley, Muir, and Waites (2000), N_2_ MAP in the absence of O_2_ increases the LT of *L. monocytogenes* in cheese up to 3 weeks and retards the growth of *L. monocytogenes*.

### 3.2 Modeling of *L. monocytogenes* growth in spinach ravioli as a function of the CSV blend and storage temperature

To predict the growth restriction of *L. monocytogenes* by adding the CSV blend in spinach ravioli with MAP, the primary growth models were generated at various temperatures. The impacts of storage temperature and the level of the CSV blend on SGR, LT, and MPD are summarized in Table 2. Adding the CSV blend at 0.3, 0.5, or 1% resulted in significant differences (*p* < .05) in the SGR values of *L. monocytogenes* in spinach ravioli, as compared with control (0%) or 0.1% (*p* < .05). Adding more than 0.3% CSV blend significantly decreased SGR from 0.15 to 0.05 log cfu/hr at 4 °C and from 0.23 to 0.06 log cfu/hr at 10 °C (*p* < .05). These results indicate that 0.3% CSV blend significantly affects SGR of *L. monocytogenes* in spinach ravioli at temperatures up to 10 °C; however, it was not sufficient to decrease SGR at ambient temperature. At 17 and 24 °C, addition of 0.5% CSV blend reduced SGR by 68 and 61%, respectively, as compared to the control.

**Table 2:** Comparison of the growth kinetics of *L. monocytogenes* in CSV blend treated spinach ravioli with MAP

		Temperature (°C)			
	Concentration of CSV (%)	4	10	17	24
SGR	0	^C^0.15 ± 0.06^a^	^C^0.23 ± 0.08^a^	^B^1.08 ± 0.37^a^	^A^1.85 ± 0.19^a^
	0.1	^C^0.09 ± 0.02^ab^	^C^0.14 ± 0.05^ab^	^B^0.94 ± 0.00^a^	^A^1.96 ± 0.06^a^
	0.3	^C^0.05 ± 0.00^bc^	^C^0.06 ± 0.00^b^	^B^0.65 ± 0.00^ab^	^A^1.64 ± 0.06^a^
	0.5	^D^0.02 ± 0.01^bc^	^C^0.06 ± 0.00^b^	^B^0.35 ± 0.01^b^	^A^0.73 ± 0.00^b^
	1	^C^0.01 ± 0.00^c^	^C^0.05 ± 0.00^b^	^B^0.27 ± 0.00^b^	^A^0.80 ± 0.01^b^
LT	0	^A^25.89 ± 2.05^b^	^B^17.70 ± 0.00^d^	^C^1.23 ± 0.03^c^	^C^1.04 ± 0.69^c^
	0.1	^A^26.30 ± 0.26^b^	^B^17.83 ± 0.02^d^	^C^1.75 ± 0.11^c^	^D^1.26 ± 0.01^c^
	0.3	^A^35.75 ± 0.02^b^	^B^25.86 ± 0.06^c^	^C^1.99 ± 0.01^c^	^D^1.20 ± 0.00^c^
	0.5	^A^56.30 ± 7.99^a^	^B^36.42 ± 0.07^b^	^C^5.16 ± 0.07^b^	^D^2.78 ± 0.01^b^
	1	NA	^A^50.21 ± 0.24^a^	^B^7.18 ± 0.00^a^	^C^6.28 ± 0.03^a^
MPD	0	^D^4.73 ± 0.00^a^	^C^7.02 ± 0.05^a^	^B^7.85 ± 0.04^a^	^A^8.09 ± 0.00^a^
	0.1	^C^5.14 ± 0.71^a^	^B^6.82 ± 0.00^a^	^AB^7.82 ± 0.08^a^	^A^8.24 ± 0.21^a^
	0.3	^D^3.35 ± 0.00^b^	^C^4.55 ± 0.00^b^	^B^4.86 ± 0.00^b^	^A^7.71 ± 0.07^a^
	0.5	^D^2.76 ± 0.04^b^	^C^3.70 ± 0.00^c^	^B^4.16 ± 0.00^c^	^A^5.75 ± 0.00^b^
	1	^C^2.47 ± 0.41^b^	^C^2.97 ± 0.00^d^	^B^4.18 ± 0.00^c^	^A^5.72 ± 0.00^b^

LT = lag time (day); SGR = specific growth rate (log/day); MPD = maximum population density (log cfu/g).

^a∼c^ Means (n = 4) ± *SD* in the same column with different superscripts are significantly different by Duncan's multiple range test at *p* < .05.

^A∼D^ Means (n = 4) ± *SD* in the same row with different superscripts are significantly different by Duncan's multiple range test at *p* < .05.

In the case of LT, the addition of 1% of CSV blend in spinach ravioli completely inhibited the growth of *L. monocytogenes* at 4 °C for 60 days storage. At 4 °C, LT of *L. monocytogenes* in spinach ravioli were extended from 25.89 (control, 0% CVS blend) to 35.75 with 0.3% CVS blend and 56.30 days with 0.5% CVS blend. At 10 °C, a significant extension of LT was still observed with the addition of 0.3 (25.86 days), 0.5 (36.42 days), and 1% (50.21% days) CSV blend compared to control (17.70 days). The LT values were significantly shortened with the increase of storage temperature. Abou‐Zeid et al. (2007) also reported that lactate‐diacetate mixture could lengthen the LT of *L. monocytogenes* only under refrigerated temperatures with pH below 6.5. In general, lag periods increase as the environmental conditions become less favorable for the growth of the pathogen. Robinson, Ocio, Kaloti, and Mackey (1998) hypothesized that lag could be determined by two parameters—namely, the amount of work to be done to adapt to a new environment and the rate at which that work can be done. Maintaining homeostasis and repairing cellular damage resulting from the antimicrobials and low temperature often require the activation of several mechanisms, which is metabolically demanding for injure cells and, therefore, leads to an extended lag phase.

The values of MPD of *L. monocytogenes* in spinach ravioli decreased significantly with the decrease of storage temperatures and the addition of the CSV blend (*p* < .05). The addition of more than 0.3% of the CSV blend in dough for spinach ravioli significantly decreased MPD at 4 °C (2.47 log cfu/g), 10 °C (4.55 log cfu/g), and 17 °C (4.86 log cfu/g), as compared to the control (*p* < .05). Thus, to achieve effective inhibition of *Listeria* growth in spinach ravioli, it is recommended to add at least 0.3% of the CVS blend in dough. In our study, when 1% CSV blend was added into spinach ravioli, MPD of *L. monocytogenes* did not exceed 3 log cfu/g for 60 days under refrigerated temperatures. At 17 and 24 °C, the behavior of *L. monocytogenes* demonstrated a significant change with 0.5 and 1% CSV blend. Other studies also reported the antimicrobial effect of the CSV blend as the function of amount. For example, Glass and Sindelar (2010), Sullivan (2012), and Schrader (2010) also reported that the 3% CSV blend reduced *L. monocytogenes* growth on meat products. In addition, the survival of other pathogens, such as *C. jejuni* and *S. Typhimurium*, on precooked chicken breasts was also controlled with 3% CSV blend (Park et al., 2014). The observed concentration‐discrepancy regarding the effectiveness of the antimicrobial could be explained by the difference in food products and food formulations during the course of experiment. The CSV blend used in the present study (up to 1%) in dough was lower than the recommended levels (3–3.5%) by the PURAC and other studies. There were significant interactions between the concentrations of the CSV blend and storage temperatures in spinach ravioli with MAP in the present study. The combined treatment of the CSV blend in dough with MAP packaging was effective at pathogen reduction, without significantly affecting the quality, and demonstrated its potential as a novel method to increase the microbial safety in ravioli, possibly other products, such as pasta and noodle, etc. Of all available options for ensuring safety, maintenance of low temperature throughout this food chain is likely to be the most effective.

The response surface models were also developed to describe the effects of the CSV blend concentration and storage temperature on SGR, LT, and MPD (Table 3). The growth kinetic values in the primary model of the present study were subjected to the response surface analysis using the SAS general linear model procedures. The developed secondary model was sufficient to predict the potential effect of storage temperature and CSV blend on *L. monocytogenes* growth in spinach ravioli. The *R* ^2^ values of SGR, LT, and MPD were .98, .94, and .93, respectively, indicating that the models effectively predicted the growth of *L. monocytogenes* in spinach ravioli with MAP.

**Table 3:** Response surface models for effect of temperature and CSV blend concentration (0–1%) on lag time (LT), specific growth rate (SGR), and maximum population density (MPD) of *L. monocytogenes* in spinach ravioli

	SGR				LT				MPD			
Parameter	Estimate	*SE*	Pr > |*t*|	*R* ^2^	Estimate	*SE*	Pr > |*t*|	*R* ^2^	Estimate	*SE*	Pr > |*t*|	*R* ^2^
Intercept	0.0479	0.1189	.6932	.98	41.3454	5.3445	<.0001	.94	4.7068	0.6134	<.0001	.93
temp	0.0172	0.0174	.3388		−3.7115	0.8064	.0005		0.2066	0.0897	.0372	
anti	−0.7709	0.2969	.0211		64.6007	13.6507	.0004		−8.5007	1.5319	<.0001	
temp × temp	0.0027	0.0006	.0004		0.0852	0.0283	.0100		−0.0012	0.0031	.6960	
anti × anti	0.9708	0.2497	.0016		−2.3074	11.8314	.8484		5.4556	1.2884	.0008	
temp ×anti	−0.0652	0.0098	<.0001		−2.6137	0.5730	.0005		−0.0237	0.0504	.6455	

In the present study, the growth of *L. monocytogenes* was not observed in MAP packed spinach ravioli with 1% CSV blend at 4 °C (Table 2). Therefore, these conditions were excluded from the development of the secondary growth model in the present study. Increased amounts of CSV blend resulted in a significant growth control of *L. monocytogenes*. The additional hurdle provided by low temperature enhanced the effect of the CSV blend as an antimicrobial agent. Conclusively, the growth of *L. monocytogenes* in spinach ravioli with MAP was safely controlled by the addition of 0.3 CSV blend at the refrigeration temperature. In summary, the models in the matrix of conditions described in the present study can be used as a tool to estimate the impact of CSV blend and temperature on the growth of *L. monocytogenes* in retail ravioli (Table 3).

### 3.3 Determination of important control factor for the growth of *L. monocytogenes* by the CART analysis

We used the CART analysis to discriminate important variables (concentration of antimicrobials, packaging method, and storage temperature) in determining the growth kinetics of *L. monocytogenes* in spinach ravioli.

**Figure 1:**
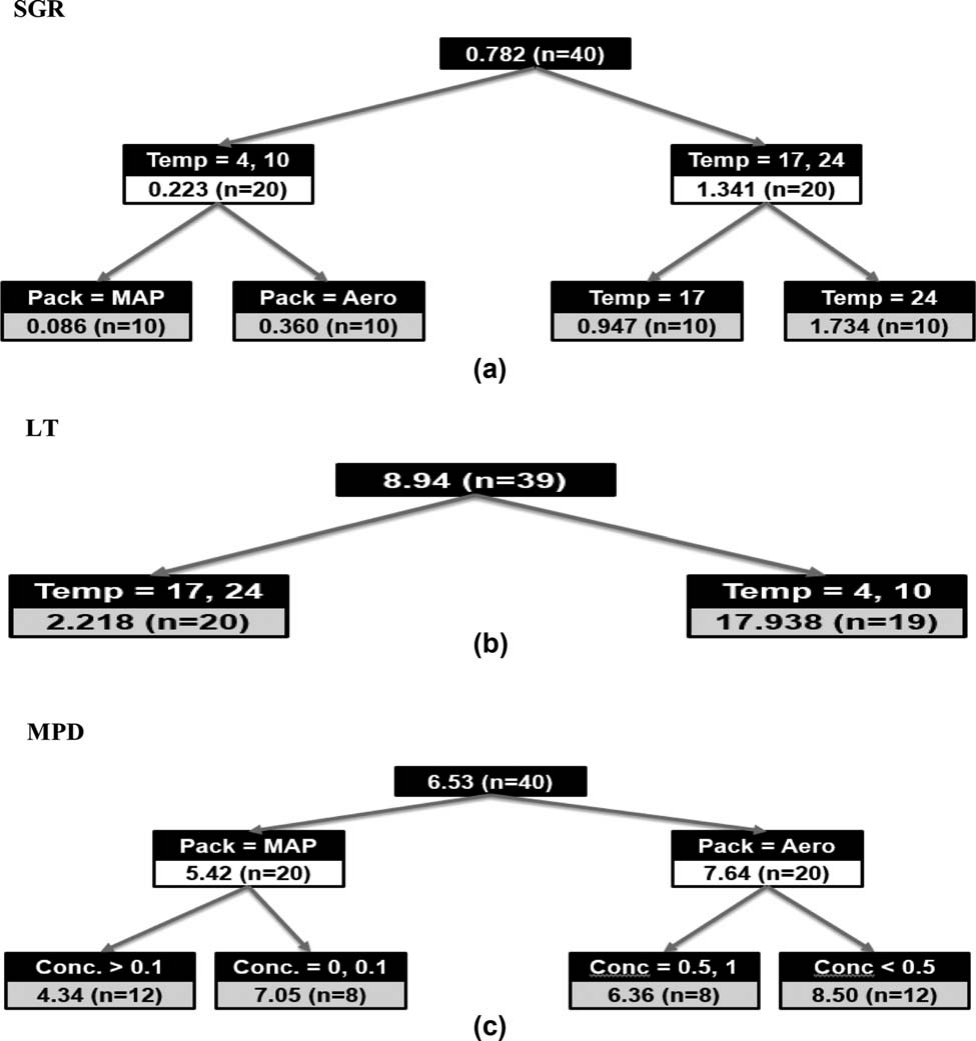
Cart analysis result of (a) SGR, (b) LT, and (c) MPD. The number in the top node indicates parameter value with the corresponding sample number in parenthesis. By following the tree structure, partitions are generated with sequential criteria of the selected factors. The root nodes are shaded in grey with final predicted SGR values for the portioned samples

Figure 1 show the results of the CART analyses using the mean absolute deviation criterion for the SGR (Figure 1b), LT (Figure 1b), and MPD (Figure 1c) values of *L. monocytogenes* in spinach ravioli. From the three variables (concentration of antimicrobials, packaging method, and storage temperature), storage temperature was the most important variable to affect SGR, followed by packaging method. Storage temperature of 4 and 10 °C discriminated SGR in spinach ravioli at a split value of SGR, 0.223 log/day, whereas SGR was sixfold greater (SGR; 1.341 log/day) at 17 and 24 °C. In the case of storage temperatures below 10 °C, packaging method discriminated SGR 0.086 and 0.360 log/day under MAP and aerobic packaging, respectively, indicating that packaging method is an important control factor for listeria growth at the refrigeration temperature, while packaging method was not an important control factor any more at ambient temperature (Figure 1a).

Also, storage temperature was the most important variables in discriminating LT. The results of the CART analysis identified that storage temperature is the only important variable to affect the LT value. At temperature less than 10 °C, LT of *L. monocytogenes* in spinach ravioli was estimated to 17.938 day, while LT at 17 °C above was approximately one‐eight shorter (2.218 day) than that at the refrigeration temperature. The results also indicated that, although packaging method and the CSV blend concentration were significant factors to affect LT, their effects were very low.

At the first split of MPD, the MPD value of *L. monocytogenes* in spinach raviolis with MAP was 5.42 log/g, while that with aerobic packaging was 7.64 log/g, which is 1.4 times higher than MAP. This result indicates that packaging method is the main control factor to affect MPD. In *L. monocytogenes* in spinach raviolis with MAP, the samples treated with above 0.1% CSV blend showed the smallest MPD value (4.34 log/g), which was 38% lower than that of raviolis without CSV blend (7.05 log/g). In the case of spinach raviolis with aerobic packaging, 0.5% concentration of CSV blend was the critical level. The MPD value of *L. monocytogenes* in spinach raviolis with less than 0.5% CSV blend with aerobic packaging was 1.3 times (8.50 log/g) higher than that of the CSV blend concentration equal to or above 0.5% (6.36 log/g). It should be noted that the threshold concentration for the MAP partition was between 0.1 and 0.3%, while that for the aerobic packaging ranged between 0.3 and 0.5%. These results indicate that low concentration of antimicrobial agent can be effective to control *L. monocytogenes* in spinach ravioli with MAP. Although the degree of importance varied, temperature was the most important factor, followed by packaging method and concentration of the CSV blend to control the growth of *L. monocytogenes* in spinach ravioli.

Consistently with the results of previous studies, our CART analysis results further emphasized that the storage at the refrigeration temperature is a critical factor in controlling *L. monocytogenes* growth in food products. The combined treatment of the CSV blend with MAP was effective in increasing *L. monocytogenes* reduction without significantly affecting the quality, and demonstrated its potential as a novel method to increase the microbial safety in ready‐to cook refrigerated ravioli.

### 3.4 Sensory evaluation

In the triangle test, 16 of 40 (45%) panelists correctly chose spinach sample treated with the 1% CSV blend that was different from the other two samples. Thus, it can be concluded with 95% confidence that not more than 50% of the population would be able to detect a difference in color, smell, or taste of spinach ravioli with 1% CSV blend. Our results also indicated that the product was well accepted by the panelists. Other studies reported that many conventional antibacterial interventions (e.g., irradiation) can result in undesirable alterations to the appearance, taste and smell of food (Gecgel, 2013) and make those foods less desirable to the consumer. However, our results showed that the organoleptic quality of spinach ravioli was not affected by application of 1% CSV blend and that no differences in taste, color, or appearance were detectable.

## 4 CONCLUSIONS

The results of this study confirm the combined efficacy of the CSV blend as a clean label/natural antimicrobial and other huddle factors, such as low temperature and MAP, on decreasing the risk of *L. monocytogenes* without significantly affecting the quality of RTC ravioli at retail market. In addition, the developed growth model for *L. monocytogenes* can predict the growth parameters of *L. monocytogenes* as a function of the CSV blend at varying concentration (0–1%) and storage temperature (4–24 °C) to determine the optimum conditions for controlling the growth of *L. monocytogenes* in spinach ravioli at retail stores, thus replace the challenge study. According to the results of our CART analysis, although the microorganism is psychrotrophic, refrigeration is the primary factor to control the rate of *L. monocytogenes* growth. In addition, MAP was an effective hurdle technique to control *L. monocytogenes* in ravioli products. According to the results of sensory evaluation, the CSV blend substantially contributes to the extension of shelf‐life of MAP spinach raviolis, delaying their spoilage while imparting a pleasant flavor.
